# MR-Guided Radiotherapy for Head and Neck Cancer: Current Developments, Perspectives, and Challenges

**DOI:** 10.3389/fonc.2021.616156

**Published:** 2021-03-19

**Authors:** Simon Boeke, David Mönnich, Janita E. van Timmeren, Panagiotis Balermpas

**Affiliations:** ^1^Department of Radiation Oncology, University Hospital and Medical Faculty, Eberhard Karls University Tübingen, Tübingen, Germany; ^2^Section for Biomedical Physics, Department of Radiation Oncology, University Hospital and Medical Faculty, Eberhard Karls University Tübingen, Tübingen, Germany; ^3^Department of Radiation Oncology, University Hospital Zurich, Zurich, Switzerland

**Keywords:** MRI, MR-guidance, IGRT (Image Guided Radiation Therapy), head and neck (H&N) cancer, adaptive radiotherapy, xerostoma, salivary gland

## Abstract

Based on the development of new hybrid machines consisting of an MRI and a linear accelerator, magnetic resonance image guided radiotherapy (MRgRT) has revolutionized the field of adaptive treatment in recent years. Although an increasing number of studies have been published, investigating technical and clinical aspects of this technique for various indications, utilizations of MRgRT for adaptive treatment of head and neck cancer (HNC) remains in its infancy. Yet, the possible benefits of this novel technology for HNC patients, allowing for better soft-tissue delineation, intra- and interfractional treatment monitoring and more frequent plan adaptations appear more than obvious. At the same time, new technical, clinical, and logistic challenges emerge. The purpose of this article is to summarize and discuss the rationale, recent developments, and future perspectives of this promising radiotherapy modality for treating HNC.

## Introduction

In recent years magnetic resonance guidance (MRg) emerged as a new promising modality within the spectrum of image-guided radiotherapy (IGRT) (1), allowing for better tumor and soft tissue visualization, repetitive imaging without additional dose exposure, target volume gating, and online plan adaptation (2). Following the first platforms with these features, including low-field MR-imaging facilities and a cobalt source (3), soon the first hybrid platforms were developed combining this image modality with a linear accelerator (MR-Linacs) (4).

At present, MR-Linacs are widely used for treating various indications and tumor localizations, e.g., stereotactic body radiotherapy (SBRT) of the upper abdomen or the lung, prostate cancer, and other pelvic targets like the rectum (5). These applications are predominantly chosen due to the obvious benefits of daily plan adaptations when including target volumes and organs at risk (OAR) with distinct inter- and intrafractional motion or anatomical changes and due to the often used hypofractionated regimens limiting the efforts of repetitive adaptations (6, 7). On the other hand, implementation of this novel technology for treating head and neck cancer (HNC) remains at its infancy, and published data about its technical and clinical applications are scarce (8) and mainly limited to MR-cobalt platforms (9). However, despite the technical and clinical challenges of HNC-radiotherapy such as long-course regimens, enhanced acute toxicity, and patient immobilization using masks compromising treatment tolerance and more complex plans with a multitude of OAR, the first research groups have already started exploiting possible benefits of MR-Linacs for this indication (10–13).

The goal of this article is to present current developments in the field of MR-guided, adaptive radiotherapy for HNC and discuss clinical benefits and difficulties of the adoption of this promising technique. For this purpose, and because of the lack of a broad consensus regarding the MRg-definition, also data and knowledge gained from MR-planning guidance before x-ray IGRT were included.

## Adaptive Treatment for Head and Neck Cancer and Potential Benefits

The concept of adaptive radiotherapy (ART) for HNC relies on accounting for potential anatomic changes during the treatment course, associated with, amongst others, tumor shrinkage, weight loss, or organ/structure migration and has been heavily exercised in the last two decades.

**Figure 1:**
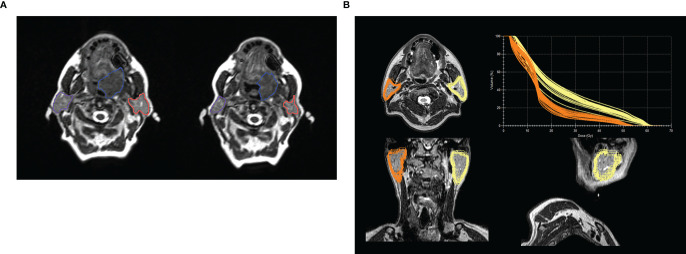
**(A)** Example of volume changes and migration of parotid glands during the course of fractionated radiotherapy at an 0.35 T MR-Linac or a large base of tongue carcinoma between treatment start (left image) and beginning of the 7th treatment week - boost (right image). Left and right parotid glands are delineated in orange and violet respectively and the gross tumor volume in blue. The volume of the left and right parotid glands decreased by 8.2 cc and 10.0 cc, respectively. The inter-parotids distance changed from 11.0 cm to 10.3 cm. **(B)** Example of a post treatment analysis for a patient treated for a hypopharyngeal carcinoma with 70 Gy in 35 fractions. Parotid glands were contoured for each daily MRI during the course of fractionated radiotherapy at a 1.5 T MR-Linac and propagated to the T2w planning MRI, with the total plan DVH for each daily delivered plan in the upper right corner, showing the variance in actual delivered dose depending on volume of the parotid gland. Averaged D_mean_ of the anatomically corrected and daily adapted plans was 24.4 Gy and 16.5 Gy for the left and right parotid glands, respectively. The D_mean_ of the reference plan was 25.9 Gy for the left and 16.7 Gy for the right parotid gland. Baseline volume was 31.0 ccm for the right and 34.5 ccm for the left parotid gland. Mean volume (range) during treatment was 30.3 ccm (29.5–32.1) and 31.4 ccm (29.1–34.7). The example was presented as a poster at the congresses of DEGRO and AIRO 2019 by Monica lo Russo, MD (20).

The original purpose of ART was to compensate for target position variability during radiotherapy in order to ensure correct dose accumulation, which led to the development of on-line 3D-imaging in the form of cone-beam-CT (CBCT) (14). Yet, most modern ART-approaches focus more on improving dose-sparing for specific OARs like the parotids (15–17). Although there is a lack of prospective clinical trials evaluating the objective benefit of ART for HNC, several dosimetric studies have been published so far, e.g., demonstrating an underestimation of the cumulative dose to the parotids when using the original non-adapted plan only, leading to increased probability for xerostomia (15, 18). Raghavan et al. were one of the first groups to demonstrate both a migration of the center of mass of the parotids, as well as a bilateral volume shrinkage in 6 HNC-patients, using an MRgRT-dedicated platform (19). An example of parotid migration and volume reduction demonstrated with the help of longitudinal imaging on the MR-Linac is shown on **Figure 1A**. An example of actual dose delivered to the parotid glands contoured offline after completion of treatment on a MR-Linac is shown in **Figure 1B** (20). Mohamad et al. showed that MRgART may be beneficial especially for swallowing related toxicities in HPV+ low risk HNC patients, especially at risk for long term toxicity due to the excellent outcome of these patients (21).

In general, the use of MRI during the course of HNC treatment is beneficial because of the superior soft-tissue contrast, thereby allowing for more precise tumor delineation and margin reduction (22). Therefore, daily online adaptive MR-guided RT could potentially be beneficial for fast responding tumors, e.g., Epstein-Barr positive nasopharyngeal cancers or HPV-positive oropharyngeal cancers (OPC) (23). Also, patients with large respiration- or swallowing induced tumor motion, like in the case of laryngeal carcinoma could benefit from MRgRT (24). But, generally, anatomical changes in the head-and-neck region are slower, e.g., caused by weight loss or target volume changes. Several studies have investigated adaptive RT for head and neck treatments, but not many studies have considered this in the presence of a magnetic field. One study in 2018 has investigated plan quality after weight loss in the presence of a magnetic field (10), showing that the current approaches of offline planning once or twice per week might be sufficient for reducing the dosimetric impact of weight changes.

Besides improved soft-tissue contrast, another advantage of MRgRT is the potential for tumor response monitoring throughout treatment without additional imaging dose (25). One study from 2016 has studied the feasibility of treatment response assessment of head and neck cancer patients using diffusion-weighted (DW) MRI on a Cobalt-60 ViewRay system (26). This study showed variation in tumor apparent diffusion coefficient (ADC) values and consistent brainstem ADC values throughout treatment, potentially allowing for early treatment response assessment. Especially DWI is a promising candidate as a prognostic imaging biomarker in HNC (25, 27–31), but with still conflicting results depending on the parameters analyzed (32). Moreover, early changes in quantitative MR parameters in OAR such as parotid glands may help to predict late toxicity like xerostomia, enabling therapeutic interventions or plan adaptations (33, 34). Thus, MR-Linacs with their capability of longitudinal DWI, may facilitate a biologically adaptive treatment, depending on therapy response for tumors and/or OARs (35).

## MR-Guidance in Head and Neck Radiotherapy: Current State of Research

Besides FDG-PET/CT, MRI has become an essential imaging modality in staging of HNC (36–39). Moreover MRI enables a better visualization of the macroscopic tumor for target volume definition and estimation/reduction of PTV margins during radical radiotherapy (40–42), as well as reduced interobserver variability (16, 18, 43–45), although prospective evaluation on primary outcome is lacking. Moreover, offline image registration remains a pitfall, if MRI is not performed in treatment position (46, 47). For treatment on the MR-Linac a simulation scan in RT position is readily available to overcome these difficulties and simultaneously offers one of the main benefits of these platforms.

Repetitive offline MR scans show, especially for HPV-associated OPC, a shrinkage already in the first weeks of therapy (48) up to a complete response in imaging in around 50% of the patients mid-treatment (49). Most of the existing data about MR-guidance in HNC treatment is in the setting of offline MRI, as online MRgRT is still a new development with only a handful of institutions treating patients with HNC on MR-Linacs and only limited data on feasibility of MRgRT in HNC published (8, 9). Tabular overview of published series or recruiting trials is provided in **Table 1**. With the above mentioned potential benefits for OAR sparing with ART (21) and the obvious advantage of daily MR-guided therapy at hand, a first prospective phase II trial for low risk HPV-associated OPC patients was initialized by the MD Anderson Cancer Center [NCT03224000 (50)]. In this trial, low risk HPV-associated OPC patients will be treated on the MR-Linac with a protocol based adaptation for the high dose volume depending on the shrinkage of the GTV. For adaptation to shrinkage of macroscopic disease an important issue may be the blurring of the tumor borders in MR-images, which is seen in studies of serial MRI during RT (48, 49). Because of this, there might be the necessity to include a GTV to CTV margin to account for these uncertainties, which need to be addressed in proper prospective clinical trials and *post hoc* analyses of the acquired imaging data with regimens not adapting the high dose target volume.

**Table 1:** Overview of published and ongoing studies on MR-linac-based adaptive radiotherapy for head and neck cancer.

First Author/PI	Year	Study design	Platform	Total patientsn	Timepoints of analysis/adaptation	Aim	Main finding/study endpoint	Relevance
Raghavan (19)	2016	Retrospective analysis	0.35 T MRI-guided tri-cobalt 60	6	Weekly	Quantify volume changes of parotid glands and GTV	Volume decrease of 31.3% (ipsilateral) and 21.8% (contralateral) and center of mass mitigation with increased dose compared to the reference plan; GTV shrinkage of 38.7%	Possibility of underestimation of dose to the parotid glands without adaptation regarding in increased risk of xerostomia
Chen (8)	2017	Prospective institutional registry	0.35 T MRI-guided tri-cobalt 60	12	No pre-planned adaptation	Feasibility of MRg-SBRT in recurrent HNC	MRg-SBRT feasible, early toxicity within expected range	Due to MR-guidance potential to reduce margins
Chen (9)	2018	Prospective institutional registry	0.35 T MRI-guided tri-cobalt 60	18	No pre-planned adaptation	Feasibility of MRgRT in HNC	MRgRT feasible in primary treatment of HNC	Non-randomized data reporting feasibility of MRgRT in HNC with toxicity in expected range
Mohamed (21)	2018	Prospective planning study	Offline MRI	5	Weeks 2, 4, 6	Adapative RT regarding GTV shrinkage in HPV+ OPC and impact on dose to OAR	GTV shrinkage of up to 100% in primary and 80% in LN; adaptive MRgRT lowers NTCP for Dysphagia and PEG dependency, no change in mean dose to parotid glands	Structured adaptive MRgRT for low risk HPV+ OPC may decrease risk for Dysphagia/PEG dependency
Bahig (50)	2018	Prospective two-stage Phase II trial	Offline MRI and Unity	15 + 60	Weekly adaptation	Adaptive RT for GTV shrinkage in HPV+ OPC with dose reduction	LRC at 6 month	Trial aiming to show safe dose reduction with adaptive MRgRT for shrinking GTV in low risk HPV+ OPC
Balermpas (13)	2019	Prospective phase II trial (NCT04242459)	MRIdian	44	Weekly adaptation	Reduce incidence of Xerostomia	n.a.	Prospective study trying to show the potential benefit of adaptive MRgRT to reduce Xerostomia in HNC; finding new prognostic imaging biomarkers

Several more prospective protocols are open for recruitment or will be opened soon to explore the role of MRgRT in HNC in various aspects: prospective basket trials, including various tumors and localizations, explore the feasibility of MRgRT depended on slots and patient burden, due to longer treatment time, noise, and claustrophobia (NCT04172753). Concerning clinical trials dedicated to HNC, the MARTHA-trial investigates potential benefits of weekly offline adaptation, narrow CTV to PTV margins and daily MRg-IGRT for reducing xerostomia in bilaterally irradiated patients over a conventionally fractionated, curative irradiation course of 7 weeks [NCT03972072 (13)]. Patient comfort and compliance will be also evaluated as secondary endpoints. Another trial will test the capability of SBRT in HNC for patients not fit for concomitant radiochemotherapy in combination with immune checkpoint inhibition (DEHART trial, NCT04477759). This is an intriguing approach for combined treatment, especially in HNC with a strong biological rationale, including the immunosensitizing effects of radiotherapy for this indication (51) or the interplay between hypoxia and immunotherapy (52). The number of running prospective trials for HNC cancer is limited so far and the existing studies do not implement identical approaches regarding the frequency and modality of adaptation, i.e., daily versus weekly, or online versus offline adaptive radiotherapy. Up to the present day, no results of prospective trials or registries have been published as a full paper but there were several presentations on congresses (1, 53–55).

## Challenges Toward Online Adaptation

Although the number of patients with HNC treated in all of the commercially available MRgRT platforms is increasing worldwide, there still exist several open questions, both in terms of physics and logistics.

One technical challenge in treatments on the MR-Linac that is also relevant in HNC, is the electron return effect (ERE), caused by the influence of the magnetic field on secondary electrons, which results in dose enhancement and attenuation at interfaces between high/low density and low/high density tissue, respectively (56). The effect is more pronounced at higher magnetic field strength. Although this effect is taken into account during plan optimization, air-tissue interfaces, common in HNC-targets, might change during the course of treatment, resulting in variation in dose deposition and risk of hotspots where beams traverse from tissue to air. A recent study investigated the robustness of treatment plans with varying sinus filling (10), and showed that more robust plans can be generated by optimizing with an empty cavity, since the optimizer will then take into account the ERE. A recent planning study including ten patients with hypopharyngeal carcinoma studied the possible effect of a 1.5 T magnetic field on plan quality and dose to OAR. Overall there had been no significant differences in plan quality or doses to OAR, if the plan is optimized for the presence of the magnetic field (57). Nevertheless, the mean and maximal dose to the skin and maximal dose to larynx and trachea was significantly higher, which needs to be critically reviewed, when assessing clinical treatment plans. Moreover, differences in homogeneity and conformity can be observed, when compared to standard VMAT plans for conventional linacs, with unknown impact on outcome or QoL and future trials might need to address these differences, like when IMRT was introduced (58).

**Figure 2:**
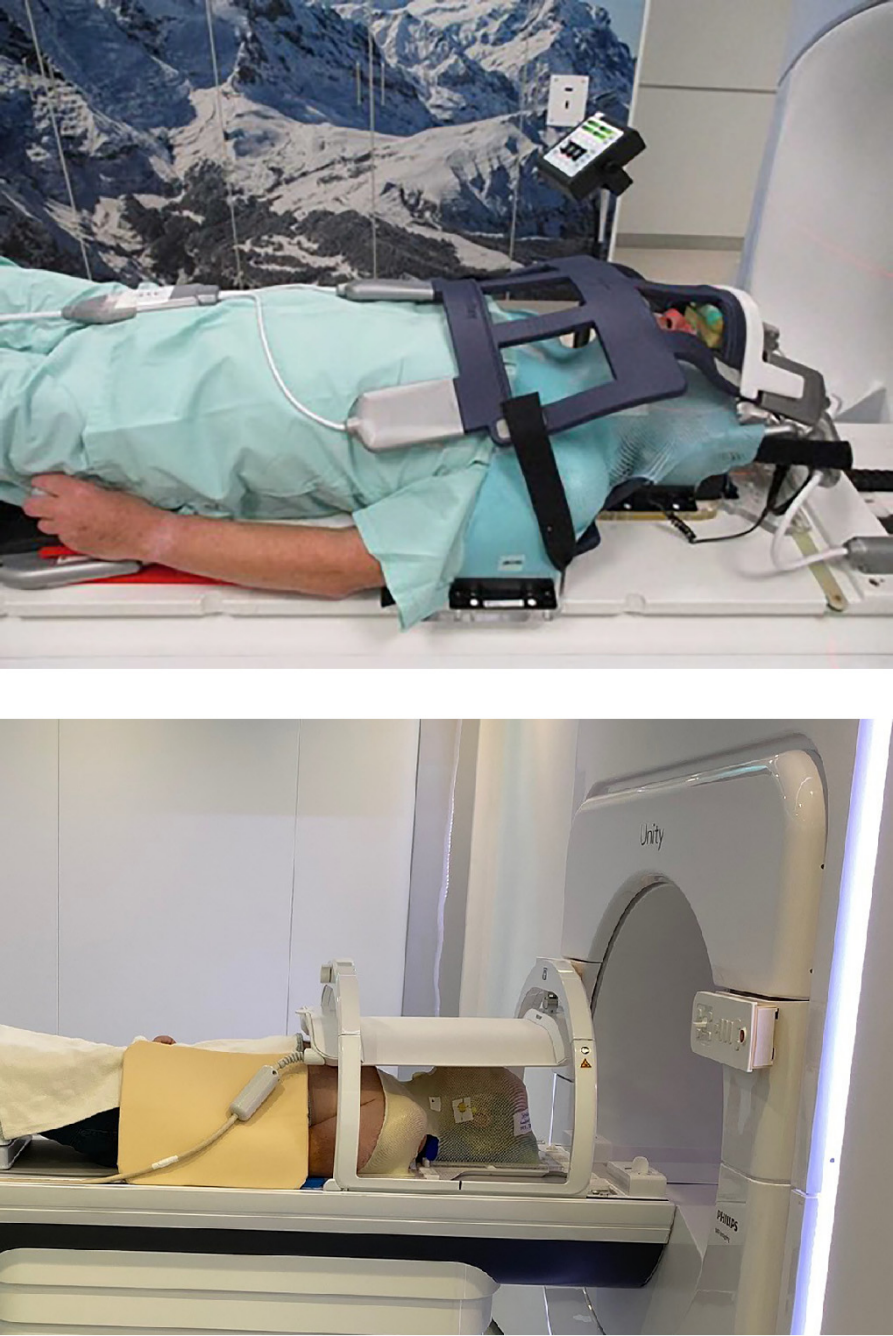
Patient positioning for MR-Linac based treatment for head and neck cancer in the two commercially available systems.

Another difficulty in head and neck treatments on the MR-Linac is the limited field of view (FOV), due to the design in which the MR gradient coil is physically split to enable a radiation window. The gap allows for maximum superior-inferior field sizes at isocenter of 22 cm for the Elekta Unity, and 28 cm for the Viewray MRIdian (59). Therefore, patients with extensive, multi-level, lymph node involvement, and/or tumors of the nasopharynx/sinonasal cavities might not be suitable for MR-Linac treatments with a single-isocenter. This, however, depends on the institutional delineation protocols and applied margins, as well on individual anatomic variations. A study from Chuter et al. (60) showed that 66.3% of the HNC-patients with a three dose-level treatment plan could be treated on the Elekta Unity, using a cranio-caudal margin of 1 cm. A reduction of this margin to 5 mm could increase the number of eligible patients by more than 15%. Another recent study showed that 6 out of 110 patients were not eligible for MR-Linac treatment with a single isocenter, including two nasopharynx patients, one oropharynx patient and three paranasal sinus patients (11). The authors stated that neutral neck position, as opposed to extended neck position, is favorable to maximise the number of patients treatable on the MR-Linac. **Figure 2** depicts a real-life patient positioning for HNC treatment on both commercially available types of MR-Linacs, implementing neutral neck position and flexible receiver coils over a thermoplastic mask.

Up to this day, a planning CT is still used for routine treatments in most institutions. However, a straightforward solution for the problem of CT/MR mismatch mentioned above would be an MR-only workflow with the problem of the missing electron density information from the CT. In the adaptive online workflow of the Elekta Unity (Elekta AB, Sweden), a contour based bulk electron density override of structures such as soft tissue, bones and air contoured on the CT and propagated to the daily MR is provided for an online reoptimization. This delineation process is time consuming and error prone and could be overcome by the means of deep learning for bone structure delineation (61–63). When using bulk electron densities for dose calculation, the CT densities of patient positioning aids cannot be used. Therefore, all positioning devices, e.g., headrests, must be contoured with sufficient detail.

Furthermore, there is concern due to the noise for HNC patients on MR-Linacs, as headphones are not compatible with standard masks, so standard foam earplugs with the maximum noise reduction of up to 37 dB is recommended. Today, there is no prospective data published to assess the possible inner ear damage, but clinical experience for HNC patients treated in our institutions so far did not show any toxicity. To the author’s knowledge, the same problem is unsolved for MR-simulations, which are routinely used in daily routine.

Finally, at the present moment, there exist several aspects that make MRgRT for HNC time consuming with currently approximately 30 min needed for applying a single fraction, and 45–50 min if online-adaptation is performed (64, 65). One of the reasons is the limited dose rate due to the larger source isocenter distance on the Unity system (5), although this is more important for SBRT with large doses per fraction compared to conventionally fractionated HNC-treatment. The dose rate of the MRIdian system is 600 cGy/minute at 90 cm SAD and such comparable to that of a conventional linac. This prolonged treatment time leads to limitations regarding the number of patients treated daily and to compliance problems over a 6 or 7 week-course of radiotherapy as is usually performed for HNC treated with curative intent. Nevertheless, some of the reasons for this time- and resource-consuming procedures could be eliminated in the near future. Both commercially available platforms (MRIdian, ViewRay Inc, Oakwood, USA and Elekta Unity, Elekta AB, Stockholm, Sweden) are only capable of delivering step-and-shoot IMRT, but there do not seem to exist any insurmountable hardware limitations for introducing dynamic MLC or VMAT (66), which will lead to significantly faster radiotherapy applications. Moreover, recent research has demonstrated that a “full” online plan adaptation does not always show significant benefits (64) and that a simple plan re-optimization is often enough for providing plans of sufficient quality (7). Applying modern developments in artificial intelligence and machine learning, in order to improve image registration and automated segmentation, could further considerably reduce time for adaptation (67).

The above facts (noise, longer treatment-time etc.) demonstrate that current practice of MRgRT is most times associated with limitations, not only of technical nature (like VMAT versus IMRT), but also with a smaller or larger compromise in terms of patient comfort. This issue becomes even more significant as most of our current treatments are applied over 6–7 weeks.

For the intriguing concept of response or biologically adaptive radiotherapy, e.g., by the means of functional imaging, several important prerequisites, like accuracy and repeatability of the measured values as well as geometrical distortions need to be taken into account. First phantom studies showed that both platforms are capable of meeting these prerequisites (24). Nevertheless, as especially the head and neck area with movement of tissue due to breathing and swallowing as well as air-tissue interfaces and the missing dedicated head and neck coils, *in-vivo* data for serial DWI on MR-Linacs acquired with the recommended procedures (68, 69) is missing (70).

## Discussion

Although MRgRT has advanced to an established modality for treating various tumor types, even for challenging tumor localizations like prostate cancer and moving targets like liver malignancies, implementation of this novel technique for irradiating HNC remains at its infancy. This article summarizes the most important rationales and obstacles behind this IGRT method so far and tries to present future directions of research in this quickly evolving field.

The possible benefits of adaptive MRgRT for HNC are obvious and have been exercised before with means of CT-scans, cone-beam-CT (CBCT) (16), or diagnostic MRI- (22, 45) and PET-imaging (71, 72) to serve as basis for adaptation during the 6-7 week treatment course. There are three main goals of adaptive RT cancer that can be more easily pursued with MRgRT as have been recently summarized by Corradini et al. (5): 1) adaptation to anatomical changes, 2) adaptation to tumor response, and 3) motion management. All of these issues are crucial for an effective and high-quality treatment of HNC and can be easily addressed with the new hybrid MR-Linac-platforms without additional dose exposure. The improved soft-tissue contrastation can provide -compared to CBCT- additional information not only about the external body contour and the tissue/air or tissue/bone interface, but also about relative interfractional changes of organs like the salivary glands or surgical flaps in the postoperative setting. Due to the better visualization and with more advanced adaptation algorithms and motion management strategies, classical irradiation masks may become obsolete potentially enhancing the patients comfort. First proof of principle for dedicated mask free radiotherapy planning for SRS in brain tumors showed good results for the mask free workflow (73). Furthermore, a daily monitoring of and quick reaction to tumor shrinkage, like in the case of viral-induced tumors will allow not only better sparing of OARs, but could pave the way for more elaborate dose-(des-)intensification and dose-painting trials (74), or even temporospatial fractionation approaches. Last but not least, the live, online, PTV-gating and cine-imaging allows for both 4D-planning and intrafractional motion monitoring to compensate for breathing or swallowing movement, an important feature in HNC, e.g., when treating glottic laryngeal cancer (75, 76). However, online motion management is not the only solution for such issues: Regarding motion-depended planning- and dosimetry uncertainties, offline-adaptation in different breathing/swallowing positions and calculation of the dosimetric impact might be an additional solution in these cases. Furthermore, exception gating could be applied in order to stop treatment in case of excessive motion (e.g., caused by coughing).

There still exist hurdles and handicaps in treatment planning and delivery, prohibiting a wider clinical use of MRgRT for HNC, with the most important ones being the lack of dynamic IMRT-approaches such as VMAT or dynamic MLC and the increased treatment delivery time with a potential higher treatment burden for the patient. Yet, technical advances are expected to solve these issues in the near future, making this innovative technique available for most HNC-patients. Until then, careful patient selection is of major importance. Patients with advanced tumors or nodal involvement, bilateral neck irradiation, target in proximity to sensible OARs and moving volumes are the most eager to benefit from MRgRT. Mathematical models to predict clinical benefit and guide slot allocation could facilitate patient selection, similar to the ones developed for proton treatment (77–79). Nevertheless, inter- and intra-fractional changes in head and neck tumors and anatomy do not usually take place as quick as, e.g., in the moving organs of the upper abdomen and as most of the times only conventional or slightly hypofractionated regimens are used, the potential additional benefits of online- over offline-adaptation should be always weighted against an extension of treatment time and compromise of the patient comfort. Until less time-consuming and more comfortable procedures are established, the decision regarding the time-point and frequency of plan adaptation has to be critically discussed, also considering the real clinical benefit. An, e.g., only weekly adaptation could be sufficient for many HN patients. In this case, the images could of course be directly acquired on the MR-Linac. Sufficient quality of these images and an MR-only planning procedure would simplify the process compared to an “external” MR-simulation with or without additional planning CT. Running and future trials should focus on possible toxicity reduction, but also on patient comfort, always involving patient reported outcomes (PROMs). Establishing novel, standardized patient positioning and immobilization devices or even treatment without masks based on the experience gathered by PROMs, as well as decision trees and standard operating procedures for the need of re-planning could facilitate a broader clinical implementation of MRgRT for head and neck cancer.

While there is still only a small number of prospective trials investigating applications of MRgRT for HNC, this is expected to increase in the next few years. Challenging fields of research could be not only the decrease of toxicity and the patient selection, but also the development of more advanced hardware, e.g., allowing for VMAT, or software, e.g., for monitor unit verification (66). Finally, the possibility to have access to daily, repetitive imaging during the whole course of radiotherapy could open completely new dimensions with respect to both functional imaging, like diffusion-weighted-MRI (80, 81), and radiomics (82) for predicting tumor response and normal tissue toxicity. This aspect becomes even more interesting through the possibility of comparison of high- (1.5 T) and low-field (0.35 T) magnetic resonance imaging provided by the different platforms.

This study has several limitations, most of all the non-systematic character of the review. Nevertheless, it is the first attempt to summarize the current stand of knowledge regarding MRgRT for the specific and challenging indication of head and neck cancer.

## Data Availability Statement

The original contributions presented in the study are included in the article/supplementary material. Further inquiries can be directed to the corresponding author.

## Author Contributions

PB designed the manuscript structure and supervised the content. SB, DM, and JT devised additional ideas, provided images, and edited the manuscript. All authors contributed to the article and approved the submitted version.

## Funding

The MRgRT program in Tübingen received funding from the German Research Council (ZI736/2-1), University of Tuebingen.

## Conflict of Interest

The authors declare that the Department of Radiation Oncology Tübingen receives within the frame of research agreements financial and technical support as well as sponsoring for travels and scientific symposia from Elekta AB (Stockholm, Sweden), TheraPanacea (Paris, France), Philips GmbH (Best, The Netherlands), Dr. Sennewald Medizintechnik GmbH (München, Germany), PTW Freiburg (Germany) and that the Department of Radiation Oncology of the Zurich University Hospital receives within the frame of research agreements funding for clinical trials from ViewRay Inc, Oakwood, USA. PB received research funding from ViewRay Inc, Oakwood, USA.
